# Dataset of the crystal structures, electrical transport properties, and first-principles electronic structures of GeTe-rich GeTe-Sb_2_Te_3_ thermoelectric materials

**DOI:** 10.1016/j.dib.2021.107462

**Published:** 2021-10-09

**Authors:** Tomohiro Oku, Hiroki Funashima, Shogo Kawaguchi, Yoshiki Kubota, Atsuko Kosuga

**Affiliations:** a Department of Physical Science, Graduate School of Science, Osaka Prefecture University, Sakai, Osaka 599-8531, Japan; b Department of Comprehensive Engineering, Kindai University Technical College, Mie, Nabari 518-0459, Japan; c Diffraction and Scattering Division, Japan Synchrotron Radiation Research Institute (JASRI), Sayo-gun, Hyogo 679-5198, Japan; d Japan Science and Technology Agency (JST), PRESTO, Kawaguchi, Saitama 332-0012, Japan

**Keywords:** Germanium telluride, First-principles calculation, Rietveld refinement, Ge defect, Synchrotron X-ray powder diffraction, Data description

## Abstract

The data presented in this article relate to the research article entitled “Superior room-temperature power factor in GeTe systems via multiple valence band convergence to a narrow energy range” [T. Oku et al., Mater. Today Phys. 20 (2021) 100484 (10.1016/j.mtphys.2021.100484)]. Polycrystalline (GeTe)*_n_*Sb_2_Te_3_ (*n* = 10, 12, 16, 20, and 24) bulk samples were prepared by melting and annealing. The Ge defect concentration of each composition was estimated from Rietveld refinement of the synchrotron X-ray powder diffraction patterns. Electrical properties, such as the electrical resistivity and Seebeck coefficient, were measured from three specimens of each composition to confirm reproducibility. Electronic-band-structure parameters and electronic density-of-states of each composition were obtained by first-principles calculations.

**Specifications Table**

**Table:** 

Subject	Materials Science
Specific subject area	crystal structure, band structure, and electrical properties of GeTe-Sb_2_Te_3_ thermoelectric materials
Type of data	Table Figure
How data were acquired	Synchrotron X-ray powder diffraction (SXRPD) patterns were acquired (BL02B2 beamline, SPring-8, Japan). Rietveld refinement (RIETAN-FP) of SXRPD patterns was used to determine crystal structure parameters. The Ge defect concentration was estimated from Rietveld refinement by fixing the Ge site occupancy at certain intervals and varying the scale factor and lattice constants. Electrical properties were measured by Seebeck coefficient and electrical resistivity measurements (ZEM-3, Advance-RIKO Inc., Japan) and Hall measurements (ResiTest8400LRJ, TOYO Corp., Japan). Thermogravimetric/differential thermal analysis (TG-DTA; TG/DTA7200, SII NanoTechnology Inc., Japan). Electronic band structure and electronic density-of-states were determined by first-principles calculations with AkaiKKR using the Korringa–Kohn–Rostoker method within coherent-potential approximation (KKR-CPA).
Data format	Raw Analyzed Filtered
Parameters for data collection	Materials: polycrystalline (GeTe)*_n_*Sb_2_Te_3_ (*n =* 10, 12, 16, 20, and 24) bulk samples Synthesis: melt at 1173 K overnight, anneal at 773 K for 10 days, water-quench SXRPD: 300 K, 400 K, 500 K, 600 K, and 700 K AkaiKKR calculation: GGA-PBE, semi-relativistic with spin-orbit interactions, high-quality *k*-mesh
Description of data collection	The crystal structure parameters, Ge defect concentration, electrical properties, electronic band structure parameters, and electronic density-of-states were determined for each composition of polycrystalline (GeTe)*_n_*Sb_2_Te_3_ (*n* = 10, 12, 16, 20, and 24). Three specimens of each composition were used for electrical property measurements. The SXRPD patterns were normalized by the largest peak for each composition.
Data source location	Institution: Osaka Prefecture University City/Town/Region: Sakai, Osaka Country: Japan
Data accessibility	With the article
Related research article	T. Oku, H. Funashima, S. Kawaguchi, Y. Kubota, A. Kosuga, Superior room-temperature power factor in GeTe systems via multiple valence band convergence to a narrow energy range, Mater. Today Phys. 20 (2021) 100484. 10.1016/j.mtphys.2021.100484

**Value of the Data**

• Information on the crystal structure and Ge defect concentration provides a deep understanding of the electronic band structure and electronic transport properties of this material system.
• These data are beneficial to researchers who are interested in the crystal structure, Ge defect concentration, band structure parameters, and electrical transport properties of GeTe derivatives.
• These data provide new insights to improve the thermoelectric properties of GeTe derivatives in terms of defect structure engineering and band engineering. These data also provide useful information on functional materials with respect to crystal structure, high-temperature stability, electronic band structure, and electronic transport properties.

## 1 Data Description

The data presented in this article are in reference to the research article entitled “Superior room-temperature power factor in GeTe systems via multiple valence band convergence to a narrow energy range” [1]. All the numerical datum referred to in this section are also available as supplementary data files.

Table 1 lists previously reported crystal structures of polycrystalline (GeTe)_*n*_Sb_2_Te_3_ bulk samples with various *n* and prepared by various heat treatments.

**Table 1:** Relationship between the crystal structure and heat treatment of polycrystalline (GeTe)*_n_*Sb_2_Te_3_ bulk materials (as previously reported).

*n* in (GeTe)*_n_*Sb_2_Te_3_	Crystal structure	Heat treatment	Authors
3 7 12 14 17	*R*3*m* *R*3*m* *R*3*m* *R*3*m* *R*3*m*	1. Anneal (500–550 °C, 2 days) 2. Water-quench	Rosenthal et al. [2]

9	*R*3*m*	1. Anneal (773 K, 3 days) 2. SPS (773 K, 5 min)	Chen et al. [3]

7 12 14 17 19	- - - *R*3*m* -	1. Anneal (1223 K, 2 h) 2. Water-quench 3. Anneal (873 K, 2/4/7 h) 4. Water quench 5. SPS (823 K, 5 min)	Xu et al. [4]

18	*Fm*$\bar{3}$*m*	6. Anneal 7. Water-quench 8. SPS	Xu et al. [5]

10 12 16 20 24	*Fm*$\bar{3}$*m* *R*3*m* *R*3*m* *R*3*m* *R*3*m*	1. Anneal (773 K, 10 days) 2. Water-quench	This work

**Fig. 1:**
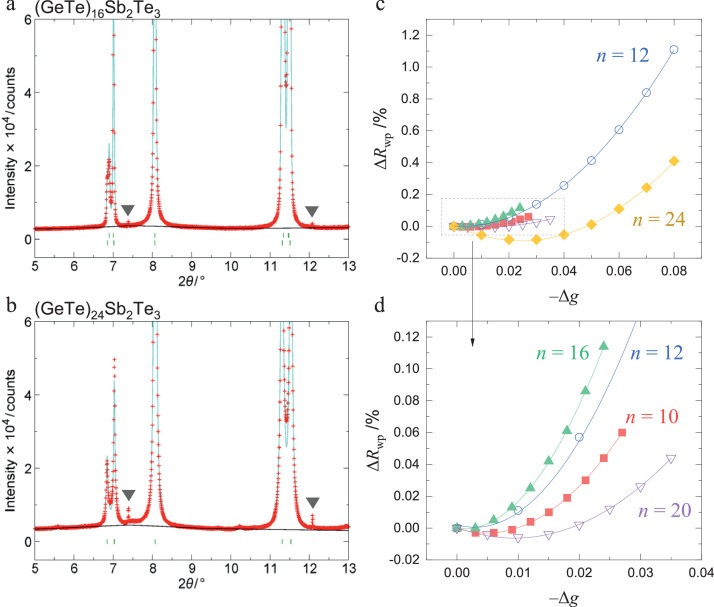
Enlarged synchrotron X-ray powder diffraction (SXRPD) patterns at 5°–13° for (GeTe)*_n_*Sb_2_Te_3_ with (a) *n =* 16 and (b) *n =* 24. The triangle symbols represent Ge peaks. (c) Amount of change in the reliability factor (Δ*R*_wp_) as a function of the amount of change in the Ge site occupancy (−Δ*g*) for (GeTe)*_n_*Sb_2_Te_3_ (*n =* 10, 12, 16, 20, and 24). Here, Δ*R*_wp_ is expressed as (*R*_wp_)_g_ − (*R*_wp_)_i_, where (*R*_wp_)_g_ represents *R*_wp_ at a certain *g*, and (*R*_wp_)_i_ represents *R*_wp_ at *g*_i_. *g* is the Ge site occupancy, Δ*g* is expressed as *g* − *g*_i_, where *g* is the Ge site occupancy, and *g*_i_ is the initial value of the Ge site occupancy. (d) Enlarged image of the dotted area in (c).

Fig. 1a and b show the enlarged SXRPD patterns of (GeTe)*_n_*Sb_2_Te_3_ (*n* = 16 and 24, respectively) from 5° to 13°. The triangle symbols represent Ge peaks. These peaks are ascribed to the presence of Ge defects of (GeTe)*_n_*Sb_2_Te_3_ (*n* = 16 and 24). Ge peaks were observed for all compositions. Fig. 1c shows the results of Rietveld analysis of (GeTe)*_n_*Sb_2_Te_3_ (*n* = 10, 12, 16, 20, and 24) to estimate the amounts of Ge defects, and Fig. 1d shows an enlarged view of the region near the origin. The horizontal axis (−Δ*g*) shows the amount of change of Ge site occupancy; Δ*g* is expressed as *g* *− g*_i_, where *g* is the Ge site occupancy and *g*_i_ is the initial value of Ge site occupancy. *g*_i_ is calculated from the Ge content in the formula of (GeTe)*_n_*Sb_2_Te_3_ (*n* = 10, 12, 16, 20, and 24); for example, in the case of *n* = 12, *g*_i_ is set as 0.8. An increase in the value of the horizontal axis (−Δ*g*) indicates that the Ge defect concentration increases. The vertical axis (Δ*R*_wp_) shows the amount of change in the reliability factor *R*_wp_, at the minimum value of which (*g*_min_), the Ge defect concentration is regarded as the best reflection of the experimental value.  Δ*R*_wp_ is expressed as (*R*_wp_)_g_ − (*R*_wp_)_i_, where (*R*_wp_)_g_ represents *R*_wp_ at a certain *g*, and (*R*_wp_)_i_ represents *R*_wp_ at *g*_i_. The curves in the figure are quadratic functions fitted by the least-squares method. Δ*R*_wp_ shows minimum values at *g* = 0.764 for *n =* 10; *g* = 0.797 for *n =* 12; *g* = 0.841 for *n =* 16; *g* = 0.860 for *n =* 20; and *g* = 0.864 for *n =* 24. This corresponds to Ge defect concentrations (*g*_i_ − *g*_min_) of 0.5% for *n =* 10; 0.3% for *n =* 12; 0.1% for *n =* 16; 0.9% for *n =* 20; and 2.4% for *n =* 24.

**Fig. 2:**
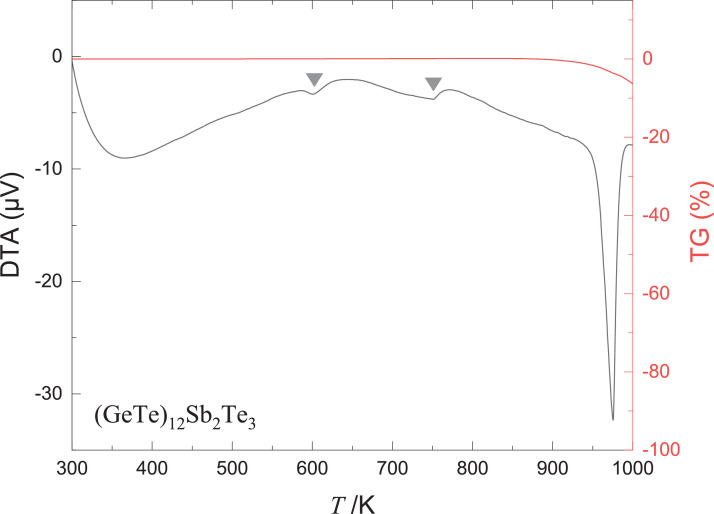
Thermogravimetric-differential thermal analysis (TG-DTA) of (GeTe)_12_Sb_2_Te_3_. The triangles indicate the peak positions of the endothermic reaction.

Fig. 2 shows the thermogravimetric-differential thermal analysis (TG-DTA) results of (GeTe)_12_Sb_2_Te_3_. Endothermic reactions occur approximately at 600 and 750 K. The melting point of (GeTe)_12_Sb_2_Te_3_ is in the 950–1000 K range.

**Fig. 3:**
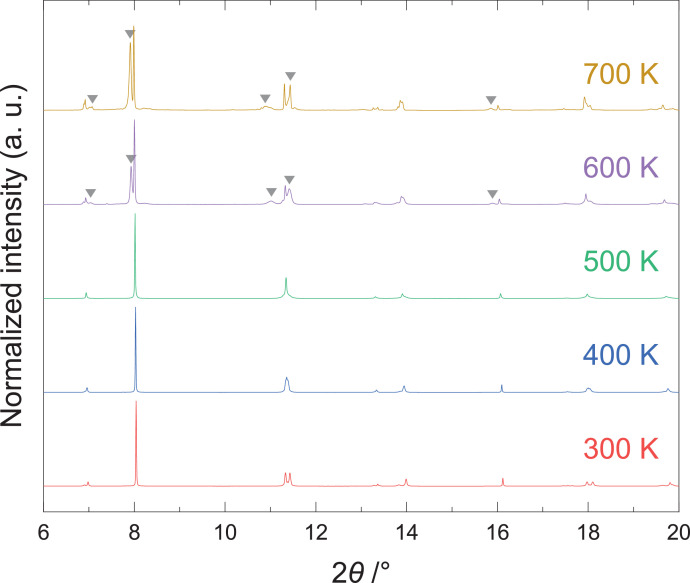
High-temperature synchrotron X-ray powder diffraction (SXRPD) patterns of (GeTe)_12_Sb_2_Te_3_. The triangles indicate peaks other than those of the main phase.

Fig. 3 shows the high-temperature SXRPD data of (GeTe)_12_Sb_2_Te_3_. These data mainly entail the rhombohedral structure (*R*3*m*) and cubic structure (*Fm*$\bar{3}$*m*) of GeTe at and below 500 K. Some peaks other than those of the main phases appeared at and above 600 K.

**Fig. 4:**
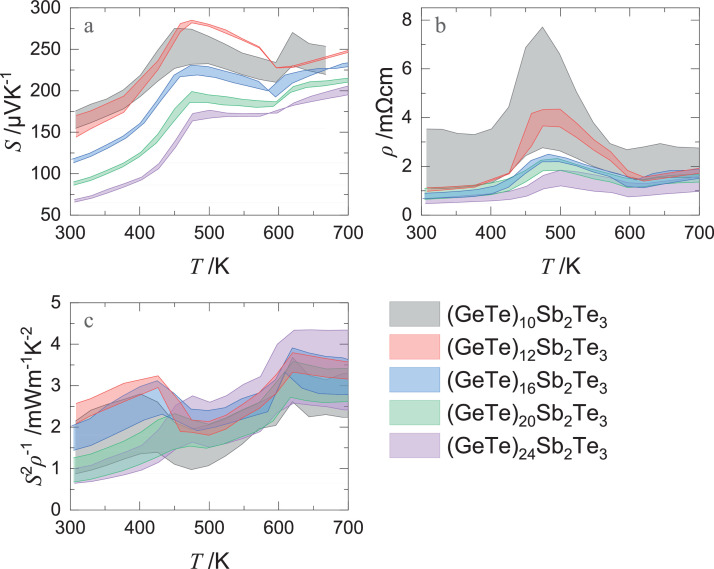
Temperature-dependence of (a) Seebeck coefficient *S*, (b) electrical resistivity *ρ*, and (c) power factor *S*^2^*ρ*^−1^, of multiple lot samples of (GeTe)*_n_*Sb_2_Te_3_ (*n =* 10, 12, 16, 20, and 24) to confirm reproducibility. The areas between the minimum and maximum measured values for multiple samples are filled in for each composition. Measurement error bars of single samples are not plotted in this figure.

Fig. 4 presents the temperature-dependent electrical properties, such as the Seebeck coefficient *S*, electrical resistivity *ρ*, and power factor *S*^2^*ρ*^−1^ of (GeTe)*_n_*Sb_2_Te_3_ (*n =* 10, 12, 16, 20, and 24). These properties were measured using three specimens per composition to confirm the reproducibility. The filled area in Fig. 4 denotes the data range from a maximum value to minimum value. Measurement errors were not plotted in this figure. The actual values of each lot sample are included in the supplementary material.

Table 2 lists the detailed band structure parameters of (GeTe)_*n*_Sb_2_Te_3_ (*n =* 10, 12, 16, 20, and 24), GeTe-10, and GeTe-12. Here, GeTe-10 and GeTe-12 have the composition of GeTe and the same lattice parameters and atomic coordinates as those of (GeTe)_10_Sb_2_Te_3_ and (GeTe)_12_Sb_2_Te_3_, respectively. The GeTe systems calculated here have a face-centered cubic (fcc) structure with space group *Fm*$\bar{3}$*m*, or rhombohedral structure with space group *R*3*m*. The cubic structures have valence band maximums (VBMs) near the Fermi level at the L band, Σ band, and Δ band. Similarly, the VBMs of the rhombohedral structures are the Z band, L band, Σ band, Σ′ band, and Δ′ band. The symbol with a prime (′), which appears in the rhombohedral structure, represents an axis in which the three-fold rotational symmetry is lost because of the reduced symmetry.

**Table 2:** Detailed band structure parameters of (GeTe)*_n_*Sb_2_Te_3_ (*n =* 10, 12, 16, 20, and 24), GeTe-10, and GeTe-12. E_VB_ (VB = L, Σ, Δ, Z, L, Σ, Σ′, and Δ′) imply the energy at the valence band maximum of each band, VB. *E*_F_ denotes the Fermi level derived from the experimental hole concentration, *n*_H_, at room temperature (approximately 300 K). *E*_g_ is the band gap energy. GeTe-10 and GeTe-12 denote the materials with the composition GeTe with the same lattice constants and atomic coordinates as those of (GeTe)_10_Sb_2_Te_3_ and (GeTe)_12_Sb_2_Te_3_, respectively.

*Fm*$\bar{3}$*m*	*E*_L_ /eV		*E*_Σ_ /eV		*E*_Δ_ /eV	*E*_F_ /eV	*E*_g_ /eV
(GeTe)_10_Sb_2_Te_3_	−0.01		−0.06		−0.19	−0.24	0.04
GeTe-10	−0.09		−0.16		−0.35	−0.28	0.18

*R*3*m*	*E*_Z_ /eV	*E*_L_ /eV	*E*_Σ_ /eV	*E*_Σ'_ /eV	*E*_Δ'_ /eV	*E*_F_ /eV	*E*_g_ /eV

(GeTe)_12_Sb_2_Te_3_	−0.16	−0.19	−0.23	−0.10	−0.24	−0.28	0.20
(GeTe)_16_Sb_2_Te_3_	−0.15	−0.15	−0.24	−0.13	−0.27	−0.32	0.21
(GeTe)_20_Sb_2_Te_3_	−0.20	−0.22	−0.34	−0.14	−0.33	−0.36	0.28
(GeTe)_24_Sb_2_Te_3_	−0.21	−0.21	−0.35	−0.13	−0.34	−0.37	0.37
GeTe-12	−0.18	−0.18	−0.29	−0.14	−0.36	−0.29	0.40

**Fig. 5:**
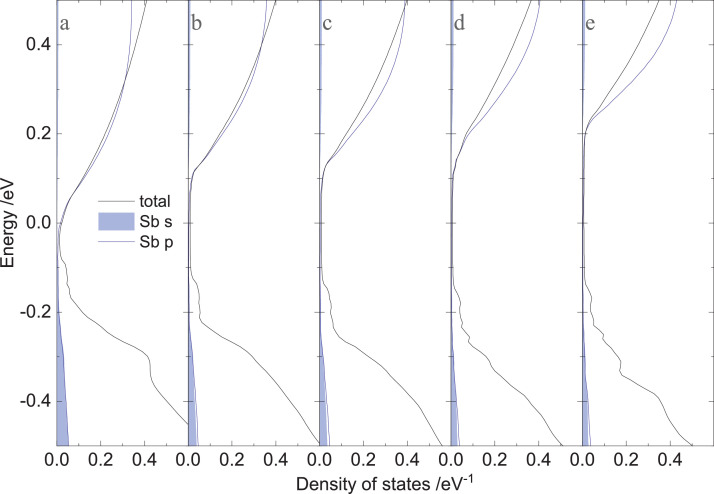
Total density of states and Sb partial density of states (*s* and *p*) near the Fermi energies of (GeTe)*_n_*Sb_2_Te_3_ with (a) *n =* 10, b) *n =* 12, c) *n =* 16, d) *n =* 20, and (e) *n =* 24.

Fig. 5 shows the total density of states and Sb partial density of states (*s* and *p*) near the Fermi energy of (GeTe)_*n*_Sb_2_Te_3_ (*n =* 10, 12, 16, 20, and 24).

## 2 Experimental Design, Materials and Methods

We synthesized polycrystalline (GeTe)_*n*_Sb_2_Te_3_ bulk samples (*n =* 10, 12, 16, 20, and 24) by melting and annealing. First, stoichiometric Ge, Sb, and Te were melted in vacuumed silica tubes at 1173 K overnight and furnace-cooled. Then, the cooled ingots were annealed at 773 K for 10 days followed by water quenching.

Synchrotron X-ray powder diffraction (SXRPD), thermogravimetric-differential thermal analysis (TG-DTA), Seebeck coefficient/electrical resistivity measurements, and Hall measurements were employed. For SXRPD, we used the BL02B2 beamline at the Japan Synchrotron Radiation Research Institute, SPring-8, with a Debye–Scherrer diffractometer [6]. Powder samples crushed from the ingots and CeO_2_ standard powder were placed in glass capillary tubes with a diameter of 0.2 mm for the refinement of X-ray wavelength. Measurements were performed at temperatures of 300, 400, 500, 600, and 700 K. We determined the crystal structures of the samples from their diffraction patterns by Rietveld refinement with RIETAN-FP [7]. Here, we also quantified the Ge defect concentration by fixing the value of the Ge site occupancy at a certain interval and varying that of the scale factor and lattice constants. TG-DTA was performed using a TG/DTA7200 instrument (SII NanoTechnology Inc., Japan) under N_2_ gas flow. The Seebeck coefficient *S*, and electrical resistivity *ρ*, were measured using a ZEM-3 instrument (Advance-RIKO Inc., Kanagawa, Japan) in the temperature range of room temperature (approximately 300 K) to 723 K. Here, the power factor *S*^2^*ρ*^−1^, was calculated using the obtained *S* and *ρ*. This measurement was performed with three samples of each (GeTe)_*n*_Sb_2_Te_3_ variant. Hall carrier density and Hall carrier mobility were measured using a ResiTest8400LRJ instrument (TOYO Corp., Tokyo, Japan) at room temperature.

We conducted first-principles calculations by using the refined structure parameters of (GeTe)_*n*_Sb_2_Te_3_ as input parameters for model-based calculations. The calculation was also performed for GeTe-10 and GeTe-12, which have the composition of GeTe and the same lattice parameters and atomic coordinates as those of (GeTe)_10_Sb_2_Te_3_ and (GeTe)_12_Sb_2_Te_3_, respectively. The programming package AkaiKKR was used for first-principles calculations, which applies the Korringa–Kohn–Rostoker method within coherent-potential approximation (KKR-CPA) [8]. The exchange and correlation potentials were constructed by the generalized gradient approximation with Perdew–Burke–Ernzerhof parameterization (GGA-PBE) [9,10]. Self-consistent field calculations were semi-relativistic, with spin-orbit interactions, in a high-quality Brillouin zone mesh. We calculated the electronic density-of-states (DOS) and evaluated the band structure parameters: *E*_VB_, energy at the valence band maximums of each band VB; *E*_F_, Fermi level; and *E*_g_, band gap energy. *E*_F_ was derived from the experimental *n*_H_ value according to the definition of carrier concentration.

## Ethics Statement

The authors declare compliance with the publication code of ethics of this journal.

## Funding

This work was supported by JST PRESTO (Grant Number JPMJPR17R4), Japan; the Thermal & Electric Energy Technology Foundation, Japan; and Tanikawa fund promotion of thermal technology, Japan.

## CRediT authorship contribution statement

**Tomohiro Oku:** Validation, Formal analysis, Investigation, Writing – original draft, Visualization. **Hiroki Funashima:** Software, Validation, Formal analysis, Resources. **Shogo Kawaguchi:** Investigation, Resources. **Yoshiki Kubota:** Investigation, Resources. **Atsuko Kosuga:** Conceptualization, Formal analysis, Writing – review & editing, Resources, Supervision, Project administration, Funding acquisition.

## Declaration of Competing Interest

The authors declare that they have no known competing financial interests or personal relationships that could have appeared to influence the work reported in this paper.

## Appendix Supplementary materials

Image, application 1
